# A plant natriuretic peptide-like molecule of the pathogen *Xanthomonas axonopodis *pv. *citri *causes rapid changes in the proteome of its citrus host

**DOI:** 10.1186/1471-2229-10-51

**Published:** 2010-03-21

**Authors:** Betiana S Garavaglia, Ludivine Thomas, Tamara Zimaro, Natalia Gottig, Lucas D Daurelio, Bongani Ndimba, Elena G Orellano, Jorgelina Ottado, Chris Gehring

**Affiliations:** 1 Molecular Biology Division, Instituto de Biología Molecular y Celular de Rosario, Consejo Nacional de Investigaciones Científicas y Técnicas, Facultad de Ciencias Bioquímicas y Farmacéuticas, Universidad Nacional de Rosario, Suipacha 531, (S2002LRK) Rosario, Argentina; 2 Consejo de Investigaciones de la Universidad Nacional de Rosario, Rosario, Argentina; 3 Department of Biotechnology, University of the Western Cape, Bellville 7535, South Africa; 4 CBRC, 4700 King Abdullah University of Science and Technology, Thuwal 23955-6900, Kingdom of Saudi Arabia

## Abstract

**Background:**

Plant natriuretic peptides (PNPs) belong to a novel class of peptidic signaling molecules that share some structural similarity to the N-terminal domain of expansins and affect physiological processes such as water and ion homeostasis at nano-molar concentrations. The citrus pathogen Xanthomonas axonopodis pv. citri possesses a PNP-like peptide (XacPNP) uniquely present in this bacteria. Previously we observed that the expression of *XacPNP *is induced upon infection and that lesions produced in leaves infected with a XacPNP deletion mutant were more necrotic and lead to earlier bacterial cell death, suggesting that the plant-like bacterial PNP enables the plant pathogen to modify host responses in order to create conditions favorable to its own survival.

**Results:**

Here we measured chlorophyll fluorescence parameters and water potential of citrus leaves infiltrated with recombinant purified XacPNP and demonstrate that the peptide improves the physiological conditions of the tissue. Importantly, the proteomic analysis revealed that these responses are mirrored by rapid changes in the host proteome that include the up-regulation of Rubisco activase, ATP synthase CF1 α subunit, maturase K, and α- and β-tubulin.

**Conclusions:**

We demonstrate that XacPNP induces changes in host photosynthesis at the level of protein expression and in photosynthetic efficiency in particular. Our findings suggest that the biotrophic pathogen can use the plant-like hormone to modulate the host cellular environment and in particular host metabolism and that such modulations weaken host defence.

## Background

Plant Natriuretic Peptides (PNPs) belong to a novel class of peptidic signal molecules that share some structural similarity with expansins [1]. While expansins are acting on the cell wall [2,3], there is no evidence that PNPs do so too. There is however an increasing body of evidence suggesting that PNPs affect many physiological responses of cells and tissues [4]. PNPs contain N-terminal signal peptides that direct the molecule into the extracellular space [5] and extracellular localization was confirmed *in situ *[6]. Recent proteomics studies have also identified the *Arabidopsis thaliana *PNP (AtPNP-A; At2g18660) in the apoplastic space [7]. *AtPNP-A *transcripts are detected in all tissues except in the embryo and the primary root [see Genevestigator [8]]. In addition, a number of PNP-induced physiological and biochemical responses including protoplast swelling [9] and the modulation of H^+^, K^+ ^and Na^+ ^fluxes in *A. thaliana *roots [10] have been reported. PNPs are also implicated in response to abiotic stresses (e.g. phosphate deprivation [11]) as well as in response to plant pathogens [12].

Surprisingly, we found a *Xanthomonas axonopodis *pv. *citri *(Xac) PNP-like protein (XacPNP) that shares sequence similarity and identical domain organization with PNPs. A significant excess of conserved residues between the two proteins within the domain previously identified as being sufficient to induce biological activity was also observed [13]. Since no significant similarity between the *X. axonopodis *pv. *citri *protein and other bacterial proteins from GenBank was detected, we firstly proposed that the *XacPNP *gene may have been acquired by the bacteria in an ancient lateral gene transfer event and speculated that this might be a case of molecular mimicry where the pathogen modulates host homeostasis to its own advantage. In addition, we have recently demonstrated that recombinant XacPNP and AtPNP-A trigger a number of similar physiological responses and made a case for molecular mimicry [14,15] where released XacPNP mimics host PNP and results in improved host tissue health and consequently better pathogen survival in the lesions.

Biotrophic pathogens like Xac rely on living host cells to be provided with nutrients. In order to fight against these pathogens, plants induce programmed cell death that is a defence mechanism aimed to limit pathogen growth. On the other hand, necrotrophic pathogens benefit from host cell death since they feed on dead tissue. It is therefore essential that plants activate the appropriate defence response according to the pathogen type. Salicylic acid (SA)-mediated resistance is effective against biotrophs, whereas jasmonic acid (JA)- or ethylene-mediated responses are predominantly against necrotrophs and herbivorous insects [16]. Several pathogens have acquired the ability to modify these plant hormone signaling and commandeer host hormonal crosstalk mechanisms as a virulence strategy (recently reviewed by [17]). For example, some *Pseudomonas syringae *strains produce a phytotoxin called coronatine (COR) [18] that structurally resembles JA derivatives [19]. Several research groups have shown that *P. syringae *employs COR to mimic JA signaling and thereby suppresses SA-mediated defence through antagonistic crosstalk [20]. Moreover, COR could suppress stomatal defence, allowing the pathogen to enter host tissue [21]. Pathogen infection has profound effects on hormonal pathways involved in plant growth and development. In that context, perturbing auxin homeostasis appears to be a common virulence mechanism, as many pathogens can synthesize auxin-like molecules. Loss of the ability to synthesize auxin-like molecules renders these pathogens less virulent [22]. Also, some pathogens deliver effector proteins that may directly impact on host auxin biosynthesis [23]. Recent works highlight the role of abscisic acid (ABA) in either promoting or suppressing resistance against various pathogens. Particularly, *P. syringae *pv. *tomato *infection dramatically induced the biosynthesis of ABA [24]. In addition, the effector protein HopAM1 aids *P. syringae *virulence by modulating ABA responses that suppress defence responses [25].

Here we report that XacPNP affects both photosynthetic parameters and the host proteome after short term exposure and discuss these findings in the light of plant-pathogen interactions. We also discuss the possible cooperation of ABA and PNP in the regulation of host homeostasis under pathogen attack.

## Results and Discussion

### Effect of XacPNP in Host Photosynthetic Efficiency and Tissue Hydration

**Figure 1:**
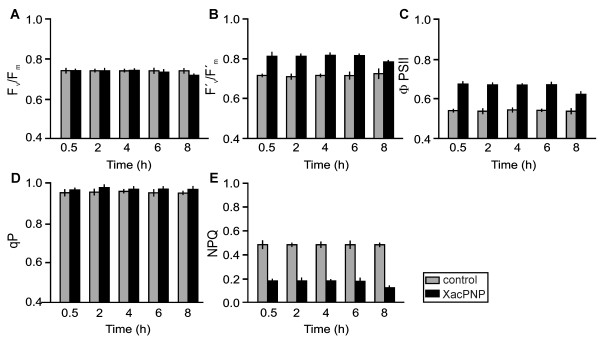
**Chlorophyll fluorescence parameters in citrus leaves treated with XacPNP**. (A) Potential quantum efficiency of PSII (F_v_/F_m_); (B) effective quantum efficiency of PSII (F'_v_/F'_m_); (C) PSII operating efficiency (ϕ_PSII_); (D) photochemical fluorescence quenching (qP) and (E) nonphotochemical fluorescence quenching (NPQ) of control and XacPNP-infiltrated citrus leaves at the times stated. The results are the mean of six replicates and error bars represent the standard deviations.

We have previously shown that XacPNP triggers a number of physiological responses similar to those caused by AtPNP-A [14] and that its presence in the citrus bacterial pathogen counteracts the reduction of host photosynthetic efficiency [26]. Thus to gain insight into the effects of XacPNP in the response on host plants, we analyzed whether this recombinant bacterial protein could modify photosynthetic performance by examining chlorophyll fluorescence parameters [27]. To this end, citrus leaves were infiltrated with 5 μM XacPNP in 50 mM Tris and chlorophyll fluorescence measured after 30 minutes, 2, 4, 6 and 8 hours. XacPNP-treated leaves showed similar values of maximum quantum efficiency of photosystem II (PSII) (F_v_/F_m_) than control leaves (50 mM Tris), indicating similar maximal intrinsic efficiency of PSII when all the centres are opened (Figure 1A). On the other hand, at a light intensity of 100 μmol quanta m^-2 ^s^-1 ^XacPNP improves both, the quantum yield of PSII photochemistry (F'_v_/F'_m_) (Figure 1B) and the PSII operating efficiency (ϕ_PSII_) and this improvement is maintained until at least 6 hours after protein infiltration (Figure 1C). The values obtained for these parameters in the presence of XacPNP were statistically different from the control leaves infiltrated with buffer at p < 0.05 and 0.001, respectively, and indicated that the efficiency of the photochemistry and linear electron transport through PSII are enhanced in response to this peptide. In contrast, no differences were observed in the photochemical quenching (qP) (Figure 1D), whereas non photochemical quenching (NPQ) showed a significant decrease in energy loss as heat as a consequence of XacPNP treatment (p < 0.01), and this is indicative of more efficient use of energy (Figure 1E). In summary, the bacterial natriuretic peptide-like protein can improve the rate of linear electron transport. However, we cannot rule out the possibility that the effect on photosynthetic efficiency could be due to secondary effects given the improved tissue condition observed in leaves infected with the wild type pathogen compared to those infected with bacteria lacking *XacPNP *[14]. Further analyses will be needed to elucidate the mechanisms and signalling pathways that lead to this effect on photosynthesis. However, we observed that the improvement in photosynthetic efficiency was maintained for some hours, suggestive of a lasting effect of this protein on the host photosynthetic machinery. Moreover, our previous results on the *XacPNP *expression in bacteria recovered from infected tissue indicates that its expression begins 24 h after infiltration and increases thereafter [14], suggesting a continuous release of the peptide to exert its function in the host plant cell. Recently, we also demonstrated that the expression of *XacPNP *in *X. axonopodis *pv. *citri *reduces the severity of reduction of key photosynthetic proteins during pathogenesis and that this effect is observed until day 6 post infiltration [26]. Therefore, all results obtained to-date suggest that this peptide improves and/or protects photosynthetic activities during pathogen attack.

PNP-dependent protoplast swelling is a well documented response and is explained by net water uptake [9,28,29]. Here we investigated the effect of XacPNP on the water status in the host plant tissue. We measured water potential in XacPNP-infiltrated leaf tissue and obtained values of -1.65 ± 0.25 MPa while for control leaves values were -2.4 ± 0.20 MPa. Since water potential gives a measure of the relative tendency of water to move from one area to another, the higher values observed for XacPNP-treated leaves point to an increased tendency of water to enter cells in the treated tissue and thus support the idea that bacterial PNP induces tissue hydration.

The physiological results presented here reinforce the idea that XacPNP is involved in host homeostasis modulation since, at a given light intensity, XacPNP-treated leaves show improved efficiency of PSII photochemistry and of the linear electron transport through PSII. The peptide also triggers a more efficient use of the energy since in treated leaves less energy is lost as heat. It is well documented that water stress produces an overall decrease of the rate of electron transport through PSII and that the photochemical efficiency of PSII decreases with the leaf water potential [30]. Water stress in agricultural plants is ameliorated by the use of cytokinin-type phytoregulators that increase the stability of the photosynthetic apparatus under such unfavourable environmental conditions [30]. Cytokinins are known to increase water influx into vacuoles, which raises the turgor pressure, which in turn opens the pores of stomata. In this way, they ensure an increased supply of carbon dioxide and increase in photosynthesis. It was recently reported [31] that over-expression of isopentenyltransferase, an enzyme that catalyzes the rate-limiting step in cytokinin biosynthesis, causes an elevation in cytokinin-dependent photorespiration, which can explain the protection of photosynthetic processes beneficial during water stress [31]. We previously demonstrated that in guard cells XacPNP causes starch degradation with a consequent rise in solute content, which in turn induces stomatal opening, causing increased in net water flux through the leaf [14]. Here we show that XacPNP can enhance plant water potential and propose that much like cytokinins, XacPNP significantly improve the performance of photosystem II through the amelioration of the leaf water status and by increasing stomata resistance. The results goes some way to establish XacPNP as a modulator of host responses particularly at the level of tissue hydration and photosynthetic efficiency, outcomes that favour biotrophic pathogen survival [14].

### Two-Dimensional Gel Electrophoretic Analysis of Protein Expression and Mass Spectrometric Identification of Induced Protein Spots

**Figure 2:**
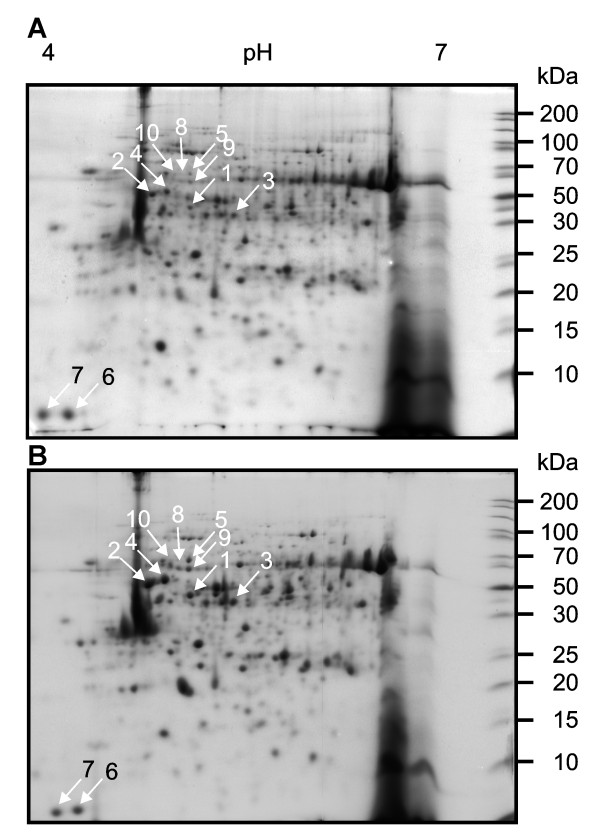
**2-DE analysis of citrus leaves proteins induced by XacPNP**. Protein profiles in 2-DE SDS-PAGE of urea-buffer extracted total soluble proteins of citrus leaves stained with Coomassie blue. Equal amounts of proteins (150 μg) were separated on 7 cm pI 4-7 linear gradient strips in the first dimension and on 12% SDS-PAGE in the second dimension. (A) citrus leaves infiltrated with Tris 50 mM solution as control; (B) citrus leaves infiltrated with 5 μM XacPNP. Proteins with significantly different expression levels between control and infected plants (p < 0.05) are indicated with white arrows and numbered. Numbers refer to protein spot numbers on Table 1. Numbers on the right indicate molecular mass in kilodalton (kDa).

Given that recombinant XacPNP causes rapid and sustained physiological changes in the host, we were interested in investigating if these changes are also reflected in alterations in the host proteome. Plants were treated with XacPNP in 50 mM Tris for 30 min and proteins were extracted for proteomics analyses. Since the buffer was required to keep XacPNP in solution, we ascertained that it did not modify photosynthetic efficiency after 30 min. Ten protein spots that showed the most reproducible increase in abundance in XacPNP treated leaves, as shown by the PDQuest analysis (Figure 2), were identified and analysed by mass spectrometry. The results are detailed in Table 1. We observed significant increases in the chloroplast proteins Ribulose-bisphosphate carboxylase (Rubisco) activase and the α-subunit of the chloroplast F1 ATP synthase. In addition, the chloroplast transcript processing enzyme maturase K also accumulated in response to XacPNP. We also noted increases in tubulin α-chain and β-tubulin 1, both of which are cytosolic.

**Table 1:** Identification of XacPNP--induced proteins with MALDI-TOF mass spectrometry

Spot n°	Protein name	Species and accession n°	Predicted MW/pI	Observed MW/pI	MOWSE Score	Match/% coverage
**1**	Rubisco activase	*Ipomea batata *ABX84141	48/8.16	40/5.4	71	9/29
**2**	Rubisco activase	*Malus x domestica *S39551	48/8.20	48/5.0	75	10/30
**3**	Rubisco activase, fragment	*Nicotiana tabacum *S25484	26/5.01	30/5.6	70	6/30
**4**	Rubisco activase alpha 2	*Gossypium hirsutum *Q308Y6	47/4.84	50/5.1	105	11/36
**5**	ATP synthase CF1 α subunit	*Citrus sinensis *YP_740460	55/5.09	60/5.3	138	14/33
**6**	Maturase K	*Alternanthera pungens *AAT28225	60/9.67	<10/4.4	77	12/37
**7**	Maturase K	*Capsicum baccatum *ABU89355	38/9.65	<10/4.4	80	10/42
**8**	Tubulin α-chain	*Prunus dulcis *S36232	49/4.92	60/5.2	86	9/30
**9**	Tubulin α-chain	*Prunus dulcis *S36232	49/4.92	55/5.3	121	11/34
**10**	β-tubulin 1	*Physcomitrella patens *Q6TYR7	50/4.82	60/5.25	156	18/44

In the following, we provide a brief characterisation of the isolated proteins, and where appropriate, a rationale for the proteomic assignment. Rubisco activase is the enzyme regulating Rubisco activity by hydrolysing ATP to promote the dissociation of inhibitory sugar phosphates, and this even at limiting CO_2 _concentration [32,33]. The increase in Rubisco activase observed would indicate a promotion of the dissociation of inhibitory sugar phosphates, and this even at limiting CO_2 _concentrations [32,33]. Such an increase in anabolism will most likely lead to net solute gain in the affected tissues.

ATP synthases are the enzymes that can synthesize ATP from ADP and inorganic phosphate. Present both in plant mitochondria and chloroplasts, ATP synthases are composed of the F_0 _and F_1 _domains [34]. ATP synthesis occurs at the β-subunit, and the α-subunit has been demonstrated to be essential for β-subunit activity [35].

Maturases are splicing factors for the plant group II introns from premature RNAs. While they generally contain three domains, the *matK *gene encodes a protein that contains only fractions of the reverse-transcriptase (RT) domain, and there is no evidence of the zinc-finger-like domain [36]. However, MATK displays the domain X (the proposed maturase functional domain) and has been assumed to be the only chloroplast gene to contain it [37]. MATK was proposed to function in the chloroplast as a post-transcriptional splicing factor [38-41]. To date, only three studies have presented evidence for the existence of a MATK protein in plants [potato (*Solanum tuberosum*, [42]), mustard (*Sinapis alba*) [43] and barley (*Hordeum vulgare*) [39]. While in barley, the identified protein product was close to the expected molecular mass for full-length MATK, the protein appears to be much smaller than expected in potato and mustard. These results indicated that MATK might be truncated in some plant species. It is noteworthy that a chloroplast ATP synthase subunit is up-regulated and this is consistent with increased metabolic activity while the MATK is indicative of splicing activities in the chloroplast. Augmented levels of MATK point to increased photosynthetic activity that is not an expected response to pathogen attack but almost certainly one beneficial to biotrophic pathogens.

Both α-tubulin (TUA) and β-tubulin (TUB), often regarded as 'housekeeping' genes, are homologous but not identical proteins that heterodimerize in a head to tail fashion to form microtubules. The latter are highly dynamic structures involved in numerous cellular processes including cell shape specification, cellular transport, cell motility, cell division and expansion [44]. In *Arabidopsis thaliana*, the TUA and TUB gene family consist of six and nine genes, respectively [45-48]. The isoforms are differentially expressed during plant development in a tissue-specific manner [47-52] and/or in response to environmental conditions [53,54]. During pathogen infection, microtubules have a role in the spread of tobacco mosaic virus from cell to cell [55]. Furthermore, it has also been described that fungal infection can lead to local microtubule depolymerisation [56]. The increased levels of tubulins may be attributed to the fact that XacPNP is inducing a hyper-hydration of the host cell, previously seen in response to Arabidopsis PNP (AtPNP-A) that is able to rapidly increase plant protoplasts volume [9]. These changes in cell volume and thus cell architecture are likely to be accompanied by changes in tubulin content. This 2-DE comparative analysis between the XacPNP and control treated leaves offered a way to identify metabolic pathways. The variation in protein expression strongly suggested that XacPNP affects metabolic activities and in particular, that after 30 min several key components of the photosynthetic apparatus are up-regulated.

### Computational systems analyses of XacPNP-responsive proteins

In order to gain further insight into PNP-dependent responses, we have identified the *A. thaliana *homologues of the proteins identified in the proteomic experiment (Table 2) and used functional annotation protocols [12,57] to infer the biological role of the homologues in the model species. A gene ontology analysis of the 50 most correlated genes, listed in Table 2 [see Additional file 1], firstly revealed that chloroplast protein encoding genes and their most correlated genes are enriched in the GO term "photosynthesis" as well as "abiotic stimuli" at level three. Secondly, the Rubisco activase gene co-expressed group is significantly enriched in the term "response to microbial phytotoxin" at level five and thirdly, the maturase K and co-expressed genes are enriched at level four for the terms "generation of precursor metabolites and energy" as well as "metabolic compound salvage". The cytosolic tubulin α-chain encoding gene and group of co-expressed genes are enriched for the terms "cellular component organization and biogenesis" at level three, "cytoskeleton organization and biogenesis" at level 5 and "microtubule-based process" at level 6. The β-tubulin 1 and co-expressed genes yielded no GO term enrichments.

**Table 2:** Homologues of the identified proteins in *A. thaliana*

Citrus protein identified	*A. thaliana *homolog^a^	*C. sinensis *protein or EST	% Identity/Similarity^b^
Rubisco activase	AT2G39730/NP_850320.1	EY668872.1	86/91
ATP synthase CF1 α subunit	ATCG00120/P56757.1	YP_740460	94/96
Maturase K	ATCG00040/NP_051040.2	CX048162.1	67/79
Tubulin α-chain	AT4G14960/NP_193232.1	CV887340.1	93/95
β-tubulin 1	AT5G62690/NP_568959.1	CV884976.1	98/100

^a^Accession numbers for *A. thaliana *homolog genes and proteins are provided.

^b^Identity and similarity between *A. thaliana *and *C. sinensis *homolog proteins.

When the co-expressed genes were analysed for common plant *cis*-elements in their promoter regions [see Additional file 1], we noted the presence of the "ABRE-like binding site motif" in the chloroplast located proteins reported here. ABRE (abscisic acid (ABA)-responsive element binding protein) [58] is a transcription factor (TF) with a role in ABA mediated responses to drought and high salt and hence homeostatic disturbances [59]. The second TF binding site in common with the group of chloroplast co-expressed genes is the CACGTG motif [60].

The stimulus response analysis in "Genevestigator" [summarised in Additional file 2A] informs that the genes encoding proteins with chloroplast function - Rubisco activase, ATP synthase CF1 α-subunit and maturase K - are down-regulated by abscisic acid (ABA). Rubisco activase and maturase K are also down-regulated by drought, which in turn down regulates tubulin α-chain and the β-tubulin 1 encoding genes. The latter two are up-regulated by the cytokinin hormone zeatin and down-regulated by the pathogen *P. syringae*.

The stimulation of maturase K and co-expressed genes is indicative for "generation of precursor metabolites and energy" as well as "metabolic compound salvage" and can presumably keep cells alive even under conditions of increased stress, i.e. pathogen attack, and is therefore advantageous to a biotroph. In addition, the co-expressed chloroplast genes with "ABRE-like binding site motifs" suggest that XacPNP participates in the drought response, presumably affecting water and/or ion movements in the host. Given that ABA has complex antagonistic and synergistic roles in plant defence [61] and down-regulates genes encoding chloroplast proteins, we propose that XacPNP antagonises ABA effects in chloroplasts. This is consistent with previous reports that showed that AtPNP-A can significantly delay ABA-caused stomatal closure [29].

We also queried "Genevestigator" to identify mutants in which the *Arabidopsis *homologues of our group of citrus genes were transcriptionally up- or down-regulated [summarised in Additional file 2B]. For the *Arabidopsis *homologues, all genes are up-regulated in the lec1-1.3 mutant. The lec (leafy cotyledon) mutants are homeotic mutants that cause defective embryonic maturation and viviparous embryos that are not insensitive to ABA but have an altered response to desiccation stress [62]. LEC transcription factors stimulate ABA levels and activate genes that repress giberellin (GA) levels, contributing to the high ABA to GA ratio characteristic of the embryonic maturation phase. High ABA levels in turn stimulate LEC to activate seed protein genes, and the reduction in GA levels might facilitate LEC activity [63]. Moreover, the phenotype of the gain-of-function mutant LEC1, in which activation of embryonic genes is augmented, is strongly enhanced by exogenously added auxin and sugars and is antagonized by cytokinin [64], thus linking auxin and sucrose levels to cell fate control and promoting cell division and embryonic differentiation. The fact that XacPNP causes starch degradation in guard cells [14] may be an indication that the increase in soluble sugars is a signal to trigger the whole photosynthetic response. We are currently in the process of conducting further analyses to determine the direct effects of XacPNP on plant carbohydrate composition and carbohydrate metabolism in plants.

It is noteworthy that ATP synthase CF1 α-subunit and maturase K are markedly down-regulated in the double loss-of-function mutant (mkk1/2). Given that the *Arabidopsis *MKK1 and MKK2 mitogen-activated protein kinases are implicated in biotic and abiotic stress responses and that the mutant has a marked phenotype in both development and disease resistance [65], we postulate that XacPNP signals, at least partly, are mediated via mitogen-activated protein kinases.

We have previously proposed that AtPNP-A may function as a component of plant defence responses given that a co-expression analysis revealed that its 25 most expression correlated genes show a significant over representation of genes annotated as part of the systemic acquired resistance [12]. It may appear quite counterintuitive that PNPs (including immunoreactant PNPs) are up-regulated in the host in response to pathogen attack [12,66], while at the same time the pathogen gains an advantage by using this molecule to its own advantage. However, it does appear that plant hormone responses are highly complex and triggered and/or modulated by specific ratios of different hormones and signaling molecules. Unbalancing such ratios will disturb optimal plant responses and this can be to the advantage of the pathogen. As an example, pathogens have been shown to increase the level of ABA and sensitivity to ABA in host plants [24], while exogenous addition of ABA to plants increases host susceptibility and this finding is consistent with the fact that ABA deficient mutants are more resistant to infection [24,67]. An explanation that was put forward is that ABA may be used by pathogens to adjust the apoplastic water status, which in turn is a critical determinant of pathogen growth [24,68]. Given that ABA and PNP cooperate with each other in a complex and tissue specific manner, it is conceivable that unbalancing the ratio of the two disturbs host homeostasis to the advantage of the pathogen. Indications for the nature of the cooperation between PNPs and ABA come from studies on stomata where they have antagonistic effects whereas PNP dependent protoplast swelling is not significantly affected by ABA [29] and while PNP signaling is critically dependent on the second messenger guanosine 3',5'-cyclic monophosphate (cGMP) [4,69], ABA signaling does not appear to be [29]. In addition, evidence for antagonistic effects of ABA and PNP was revealed by transcriptomics analyses in Arabidopsis thaliana [8] that show a >1.5 fold increase in transcript accumulation of AtPNP-A (AT2G18660) in *aba1-1 *and *aba1-1.1 *plants deficient in ABA synthesis due to a mutation in the zeaxanthin epoxygenase encoding gene. There is also a strong indirect link between ABA and PNP; ABA suppresses salicylic acid (SA) biosynthesis [67,70] and SA in turn has a marked effect on AtPNP-A transcript accumulation in Arabidopsis. In mutants with elevated SA levels (cpr5 and mpk4) AtPNP-A is markedly up-regulated (>2 fold) and conversely, in the SA deficient mutant nahG AtPNP-A transcript levels are down (>4 fold) [12]. In summary, our results suggest a role for XacPNP as an effector protein that disturbs host homeostasis to the advantage of the pathogen.

## Conclusions

We have provided experimental evidence that XacPNP present in the citrus canker pathogen is able to modify the host proteome and mainly affects proteins essential for photosynthesis and in particular photosynthetic efficiency. Gene ontology analysis as well as stimulus responses and mutant analysis suggest that these proteins might in some instances function as antagonists of ABA, while inducing similar responses to those observed with cytokinin. None of the XacPNP responsive proteins identified to date is related directly to defence responses, lending support to the idea that XacPNP functions as modulator of host homeostasis. Finally, considering that *X. axonopodis *pv. *citri *is a biotroph and not a free-living pathogen and the only known bacteria in which PNP is present, we propose that the role of XacPNP during the infection process is to maintain host cellular conditions favourable for bacterial survival.

## Methods

### Synthesis of Recombinant XacPNP

The region coding for the mature XacPNP protein was inserted into pET28a vector (Novagen, USA) and expressed in *E. coli *as an His-tag N-terminal fusion protein. Briefly, *XacPNP *was amplified by PCR using this pair of oligonucleotides: NPNPB (5' ATCA**GGATCC**GACATCGGTACAATTAGTT 3') and CPNPH (5' ATAC**AAGCTT**TTAAATATTTGCCCAGGGCG 3'), bearing *Bam*HI and *Hind*III restriction sites, respectively. After sequencing and digestion, the PCR product was ligated to the same sites in pET28a. *E. coli *BL21(DE3)pLys cells transformed with this plasmid were grown in LB medium containing antibiotics at 37°C to an absorbance of 0.8 at 600 nm. Protein expression was induced by adding 0.1 mM IPTG and incubation continued for an additional 3 h period at 30°C. Then, cells were harvested, and resuspended in 50 mM Tris-HCl pH 8.0, 150 mM NaCl, 5 mM MgCl_2_, 10 mM imidazole and 1 mM phenylmethylsulfonil fluoride. After disruption of the bacterial cells by sonication, lysates were clarified by centrifugation and proteins purified using Ni-NTA agarose resin (QIAGEN) as recommended by the manufacturer. Firstly, 1 mL of 50% Ni-NTA slurry was loaded onto a column and equilibrated with 4 mL of equilibration buffer (50 mM Tris-HCl pH 8.0, 150 mM NaCl and 10 mM imidazole). Subsequently, the clarified lysate was passed through the resin and washed twice with 4 mL of wash buffer (50 mM Tris-HCl pH 8.0, 150 mM NaCl and 10 mM imidazole). Proteins were eluted four times with 1 mL elution buffer (50 mM Tris-HCl pH 8.0, 150 mM NaCl and 200 mM imidazole) and the purification was verified by SDS/PAGE. The protein was dialized overnight against 50 mM Tris-HCl pH 8.0 and 150 mM NaCl at 4°C.

### Determination of Physiological Parameters

*Citrus sinensis *plants were grown in a growth chamber in incandescent light at 28°C with a photoperiod of 14 h. Three leaves from three different plants were infiltrated with XacPNP at 5 μM diluted in Tris 50 mM and as a control, leaves were infiltrated with Tris 50 mM. At 30 min, 2, 4, 6 and 8 hours post infiltration chlorophyll fluorescence parameters were measured using a portable pulse amplitude modulation fluorometer (Qubit systems Inc., Ontario, Canada) connected to a notebook computer with data acquisition software (Logger Pro3 Version). The minimal fluorescence level (F_o_) in the dark-adapted state was measured when only the LED light was turned on. The output from the LED light is insufficient to drive photosynthesis and does not disturb the dark-adapted state. The maximal fluorescence level in the dark-adapted state (F_m_), the fluorescence emission from leaf adapted to actinic light (F') and the maximal fluorescence level during illumination (F'_m_) were measured by a 0.8 s saturating pulse at 5000 μmol m^-2 ^s^-1^. *F*_m _was measured after 30 min of dark adaptation. F'_m _was measured with actinic light source of photon flux density (PPFD) 100 μmol m^-2 ^s^-1^. The minimal fluorescence level during illumination (F'_o_) was calculated from measured values of F_o_, F_m _and F'_m_. Variable fluorescence yield was determined in dark-adapted (F_v _= F_m _- F_o_) and in light-adapted (F'_v _= F'_m _- F'_o_) states. Photosynthetic parameters: potential (F_v_/F_m_) and effective (F'_v_/F'_m_) quantum efficiency of PSII, PSII operating efficiency {ϕ_PSII _= [(F'_m _- F')/F'_m_]}, photochemical qP = [(F'_m _- F')/(F'_m _- F'_o_)] and nonphotochemical NPQ = [(F_m _- F'_m_)/F'_m_] fluorescence quenching were calculated as described [27] and analyzed with one-way ANOVA. Leaf water potential (ψ_w_) was measured by the isopiestic thermocouple psychometric technique (Dew Point Microvoltmeter HR-33T, Wescor, USA). For this variable, an average of 10 samples (10 leaves) were taken.

### Plant Treatment and Protein Extraction

Protein extracts from three leaves infiltrated with 5 μM XacPNP in 50 mM Tris as well as control leaves infiltrated with 50 mM Tris both for 30 minutes were prepared by pulverization of leaves in liquid nitrogen followed by re-suspension in 50 mM Hepes-KOH buffer pH 7.5, 330 mM sorbitol, 5 mM sodium ascorbate, 2 mM EDTA, 1 mM MgCl2, 1 mM MnCl2 and 0.33 mM PMSF in a 1:2 ratio. The samples were centrifuged at 12000 × g at 4°C, for 20 min and soluble proteins were precipitated with 10% trichloroacetic acid (TCA) in acetone. Precipitated proteins were collected by centrifugation at 13400 × g for 10 min at 4°C. The pellet was washed three times with ice-cold 80% acetone by centrifuging at 13400 × g for 10 min per wash. The pellet was then air dried at room temperature and resuspended in urea buffer (9 M urea, 2 M thiourea and 4% 3- [(3-Cholamidopropyl)dimethylammonio]-1-propanesulfonate (CHAPS)] for at least 1 h with vigorous vortexing at room temperature. Protein content of total soluble protein was estimated by a modified Bradford assay using BSA as standard [[71]].

### Two-dimensional (2-DE) Gel Electrophoresis

Soluble protein samples (150 μg) were mixed with 0.8% (w/v) dithiothreitol (DTT), 0.2% (v/v) ampholytes pH 3-10 (BIO-RAD, Hercules, CA), 0.002% bromophenol blue and the volume was adjusted to 125 μL using urea buffer. The samples were then used to passively rehydrate linear 7 cm IPG strips, pH range 4-7 (BIO-RAD) overnight at room temperature. The strips were subjected to isoelectric focusing (IEF) using the Ettan™IPGphor II™ (GE Healthcare, Amersham, UK), in a step wise programme for a total of 3,700 Vhrs at 20°C. Prior to the second dimension, the strips were equilibrated twice for 10 min with gentle shaking in an equilibration buffer (6 m urea, 2% (w/v) SDS, 0.05 m Tris-HCl, pH 8.8 and 20% (v/v) glycerol) firstly containing 1% (w/v) DTT and then 2.5% (w/v) iodoacetamide. The strips were then loaded to 12% SDS-PAGE gels and electrophoresed at 120 V until the bromophenol blue dye reached the bottom of the gel plates (about 90 min). The gels were stained with Coomassie Brilliant Blue, imaged with the PharosFX™ plus molecular imager scanner (BIO-RAD) and analysed using the PD-Quest software (BIO-RAD). Ten spots that showed reproducible induced expression as determined by the T-test from PD-Quest (p < 0.05) were selected for mass spectrometry analysis.

### In-Gel Trypsin Digestion and Mass Determination

Spots of interest were excised manually and transferred into sterile microcentrifuge tubes. The gel pieces were washed twice with 50 mM ammonium bicarbonate for 5 min each time and a third time for 30 min, vortexing occasionally. The gel pieces were then destained two times with 50% (v/v) 50 mM ammonium bicarbonate and 50% (v/v) acetonitrile for 30 min, vortexing occasionally. The gel pieces were dehydrated with 100 μL (v/v) acetonitrile for 5 min, and then completely dessicated using the Speed Vac SC100 (ThermoSavant, Waltham, MA, USA). Proteins were in-gel digested with approximately 120 ng sequencing grade modified trypsin (Promega, Madison, WI, USA) dissolved in 25 mM ammonium bicarbonate overnight at 37°C. The protein digestion was stopped by adding 50-100 μL of 1% (v/v) trifluoroacetic acid (TFA) and incubating 2-4 h at room temperature before storage at 4°C until further analysis.

Prior identification, the samples were cleaned-up by reverse phase chromatography using ZipTip_C18_™ (Millipore, Billerica, MA, USA) pre-equilibrated first in 100% (v/v) acetonitrile and then in 0.1% (v/v) TFA and eluted out with 50% (v/v) acetonitrile. One microlitre from each sample was mixed with the same volume of α-cyna-hydroxy-cinnamic acid (CHCA) matrix and spotted onto a MALDI target plate for analysis using a MALDI-TOF mass spectrometer, the Voyager DE Pro Biospectrometry workstation (Applied Biosystems, Forster City, CA, USA) to generate a peptide mass fingerprint. All MALDI spectra were calibrated using sequazyme calibration mixture II containing angiotensin I, ACTH (1-17 clip), ACTH (18-39 clip), ACTH (7-38 clip) and bovine insulin (Applied Biosystems). The NCBI and MSDB peptide mass databases were searched using MASCOT http://www.matrixscience.com/search_form_select.html with 100 ppm accuracy and oxidation as variable modification selected. Only proteins identified with bioinformatics algorithm MOWSE scores of 70 and above were considered as positive hits.

## Abbreviations

PNP: plant natriuretic peptide; XacPNP: *Xanthomonas axonopodis *pv.*citri *PNP-like protein; PSII: photosystem II; GO: gene ontology; ABA: abscisic acid; ABRE: ABA-responsive element; TF: transcription factor; GA: giberellin; PR: pathogenesis related protein; MALDI-TOF: matrix assisted laser desorption/ionisation time-of-flight; MOWSE: molecular weight search.

## Authors' contributions

The project was conceived and designed by NG, EGO, BN, JO and CG. Proteomic analyses were performed by BSG and LT and data analyzed by BSG, LT, BN and CG. Chlorophyll fluorescence was measured by BSG, TZ and LDD. BSG measured water potentials. The manuscript was written by CG, NG and JO. All authors read and approved the final manuscript.

## Supplementary Material

Additional file 1 **GO and promoter analysis of Arabidopsis thaliana homologues of the proteins identified in the proteomics assay**. List of significantly enriched GO terms associated with the identified proteins expression correlated genes in FatiGO+. Promoter analysis for common transcription factors sites using Athena. Click here for file

Additional file 2 **Stimulus and mutants analysis of Arabidopsis thaliana homologues of the proteins identified in the proteomics assay**. (A) Stimulus response analysis in Genevestigator and (B) Identification of mutants in which the Arabidopsis homologues of the identified citrus proteins encoding genes were transcriptionally up- or down-regulated. Click here for file
