# Supplementary material for: A plant natriuretic peptide-like molecule of the pathogen Xanthomonas axonopodis pv. citri causes rapid changes in the proteome of its citrus host
Source: BMC Plant Biol. 2010 Mar 21;10:51. doi: 10.1186/1471-2229-10-51 (PMC2923525; doi:10.1186/1471-2229-10-51)
Supplement: Additional file 1 — GO and promoter analysis of Arabidopsis thaliana homologues of the proteins identified in the proteomics assay. List of significantly enriched GO terms associated with the identified proteins expression correlated genes in FatiGO+. Promoter analysis for common transcription factors sites using Athena. [file 1471-2229-10-51-S1.PDF]

Additional file 1: GO and promoter analysis of *Arabidopsis thaliana* homologues of the proteins identified in the proteomics assay.

<u>FatiGO+ and Athena results (top 50 up-regulated including target gene)</u>

1. At2g39730 (2 probes) Rubisco activase

Table 1.1: FatiGO+ analysis of **At2g39730** co-regulated genes-probe ID: 15949_at
Genes in list1 used in analysis: 51
Genes in list2 used in analysis: 28763

GO term	Level	Adjusted p value
Photosynthesis	3	8.18E-34
generation of precursor metabolites and energy	4	1.73E-07
photosynthesis, light reaction	5	1.3E-18
regulation of photosynthesis	5	2.85E-05
electron transport	5	3.74E-03
NADP regeneration	5	6.36E-03
response to microbial phytotoxin	5	9.65E-03
oxidoreduction coenzyme metabolic process	5	1.68E-02
photosynthetic electron transport	6	1.9E-07
regulation of generation of precursor metabolites and energy	6	4.43E-06
photosynthesis, light harvesting	6	7.39E-06
regulation of protein stability	6	1.16E-05
photosynthetic electron transport in photosystem I	7	1.64E-07
regulation of photosynthesis, light reaction	7	2.76E-06
photosynthesis, light harvesting in photosystem I	7	8.58E-06
protein stabilization	7	8.58E-06
pyridine nucleotide metabolic process	7	3.8E-03
photosystem I stabilization	8	9.48E-06
nicotinamide metabolic process	8	1.97E-03
NADP metabolic process	9	9.04E-03

Table 1.2: Common TF sites of At2g39730 co-regulated genes (probe ID-15949_s_at) (Athena Results)

TF sites	P value	#P	#S
ABRE-like binding site motif	$<10^{-8}$	25	52
ACGTABREMOTIFA2OSEM	$<10^{-7}$	20	33
CACGTGMOTIF	$<10^{-6}$	20	48
SV40 core promoter motif	$<10^{-4}$	18	19
Ibox promoter motif	$<10^{-3}$	25	46
MYB4 binding site motif	0.0106	35	75
MYB1AT	0.0168	38	107

#P: number of genes containing TF sites
#S: number of TF site occurrence within gene promoters

Table 1.3: FatiGO+ analysis of **At2g39730** co-regulated genes -probe ID: 245061_at
Genes in list1 used in analysis: 51
Genes in list2 used in analysis: 28755

GO term	Level	Adjusted p value
Photosynthesis	3	1.35E-41
response to abiotic stimulus	3	1.41E-02
generation of precursor metabolites and energy	4	5.07E-13
response to radiation	4	7.61E-04
metabolic compound salvage	4	4.9E-03
Photosynthesis, light reaction	5	4E-18
response to light stimulus	5	2.23E-03
Electron transport	5	4.25E-03
photorespiration	5	4.25E-03
nucleotide metabolic process	5	2.59E-02
coenzyme metabolic process	5	3.3E-02
Regulation of photosynthesis	5	3.3E-02
Response to cold	5	4.92E-02
Alcohol catabolic process	5	4.92E-02
Photosynthetic electron transport	6	1.16E-07
Photosynthesis, light harvesting	6	8.99E-06
Nonphotochemical quenching	6	1.36E-03
Regulation of generation of precursor metabolites and energy	6	3.53E-02

Photosynthesis, light harvesting in photosystem I	7	2.12E-05
photosynthetic electron transport in photosystem I	7	2.12E-05
regulation of photosynthesis, light reaction	7	4.6E-02

Table 1.4: Common TF sites of At2g39730 co-regulated genes (probe ID- 245061_at) (Athena Results)

**TF sites**	**P value**	**#P**	**#S**
ABRE-like binding site motif	$<10^{-9}$	34	71
ACGTABREMOTIFA2OSEM	$<10^{-9}$	30	48
GBOXLERBCS	$<10^{-9}$	16	24
CACGTGMOTIF	$<10^{-8}$	24	60
Ibox promoter motif	$<10^{-6}$	35	57
MYB1AT	$<10^{-5}$	50	129
CCA1 binding site motif	$<10^{-3}$	23	27
MYB4 binding site motif	0.0012	43	88

Table 1.5: TF sites of At2g39730 (probe ID-15949_s_at) (Athena Results)

**TF sites**	**P value**	**#P**	**#S**
ABRE-like binding site motif		1	2
UPRE1AT	$<10^{-3}$	1	1
CACGTGMOTIF		1	2
SV40 core promoter motif		1	1
Ibox promoter motif		1	1
BoxII promoter motif		1	1
MYB1AT		1	3
CARGCW8GAT		1	8
GAREAT		1	1
MYCATERD1		1	1
T-box promoter motif		1	1
AtMYC2 BS in RD22		1	1
DRE core motif		1	1
GBF 1/2/3 BS in ADH1	0.0133	1	2
TATA-box motif		1	1

#P: number of genes containing TF sites
#S: number of TF site occurrence within gene promoters

2. AtCg00120 (1 probe) ATP synthase CF1 α subunit

Table 2.1: FatiGO+ analysis of **AtCg00120** co-regulated genes
Genes in list1 used in analysis: 51
Genes in list2 used in analysis: 28755

**GO term**	**Level**	**Adjusted p value**
Photosynthesis	3	3.38E-34
response to abiotic stimulus	3	8.95E-03
generation of precursor metabolites and energy	4	2.05E-11
Response to temperature stimulus	4	7.43E-03
response to radiation	4	1.19E-02
Carbon utilization by fixation of carbon dioxide	4	2.63E-02
Photosynthesis, light reaction	5	3.17E-15
Response to cold	5	2.72E-03
Electron transport	5	1.54E-02
response to light stimulus	5	2.5E-02
nucleotide metabolic process	5	4.3E-02
Photosynthesis, light harvesting	6	2.33E-05
Photosynthetic electron transport	6	2.33E-05
Nonphotochemical quenching	6	1.95E-03
Nucleoside triphosphate metabolic process	6	3.33E-02
Photosynthesis, light harvesting in photosystem I	7	3.66E-05
photosynthetic electron transport in photosystem I	7	3.66E-5
Nucleoside triphosphate biosynthetic process	7	3.05E-02
Purine nucleoside triphosphate metabolic process	7	3.05E-02
Purine ribonucleotide metabolic process	7	4.32E-02
Purine nucleotide biosynthetic process	7	4.36E-02

Table 2.2: Common TF sites of AtCg00120 co-regulated genes (probe ID- 245024_at) (Athena Results**)**

**TF sites**	**P value**	**#P**	**#S**
ABRE-like binding site motif	$<10^{-9}$	31	64
ACGTABREMOTIFA2OSEM	$<10^{-9}$	26	43
ABRE binding site motif	$<10^{-9}$	16	26
ABFs binding site motif	$<10^{-9}$	15	23
GBOXLERBCS	$<10^{-9}$	13	21
CACGTGMOTIF	$<10^{-6}$	21	54
Ibox promoter motif	$<10^{-5}$	32	50

3. AtCg00040 (1 probe) Maturase K

Table 3.1: FatiGO+ analysis of **AtCg00040** co-regulated genes
Genes in list1 used in analysis: 51
Genes in list2 used in analysis: 28755

GO term	Level	Adjusted p value
Photosynthesis	3	5.63E-26
response to abiotic stimulus	3	8.95E-03
generation of precursor metabolites and energy	4	4.33E-06
Carbon utilization by fixation of carbon dioxide	4	1.18E-03
response to radiation	4	2.77E-03
Metabolic compound salvage	4	7.68E-03
response to temperature stimulus	4	4.67E-02
Photosynthesis, light reaction	5	1.19E-12
Carbon utilization	5	3.33E-03
response to light stimulus	5	7.54E-03
photorespiration	5	7.54E-03
Response to cold	5	2.43E-02
Photosynthesis, light harvesting	6	7.07E-05
Photosynthetic electron transport	6	2.76E-03
Nonphotochemical quenching	6	2.76E-03
Photosynthesis, light harvesting in photosystem I	7	3.66E-05
photosynthetic electron transport in photosystem I	7	1.42E-02

Table 3.2: Common TF sites of AtCg00040 co-regulated genes (probe ID- 245048_at) (Athena Results)

TF sites	P value	#P	#S
ABFs binding site motif	$<10^{-9}$	14	18
GBOXLERBCS	$<10^{-9}$	13	17
ABRE binding site motif	$<10^{-8}$	15	21
ABRE-like binding site motif	$<10^{-7}$	26	51
ACGTABREMOTIFA2OSEM	$<10^{-7}$	22	36
CACGTGMOTIF	$<10^{-4}$	18	42
MYB1AT	$<10^{-3}$	47	123
MYB4 binding site motif	$<10^{-3}$	43	91

4. At4g14960 (2 probes) Tubulin α-chain

Table 4.1: FatiGO+ analysis of **At4g14960** co-regulated genes -probe ID: 245270_at
Genes in list1 used in analysis: 51
Genes in list2 used in analysis: 28755

GO term	Level	Adjusted p value
Cellular component organization and biogenesis	3	1.45E-02
Cytoskeleton organization and biogenesis	5	7.83E-04
Microtubule-based process	6	3.38E-05

Table 4.2: Common TF sites of At4g14960 co-regulated(probe ID-245270_at) (Athena Results**)**

TF sites	P value	#P	#S
DRE core motif	0.0051	17	21
AtMYC2 BS in RD22	0.0158	22	34
MYCATERD1	0.0158	22	34
ARF binding site motif	0.0489	21	23
Ibox promoter motif	0.0499	22	33

Table 4.3: FatiGO+ analysis of **At4g14960** co-regulated genes -probe ID: 261639_at
Genes in list1 used in analysis: 51
Genes in list2 used in analysis: 28755

**NO SIGNIFICANT TERMS**		

Table 4.4: Common TF sites of At4g14960 (probe ID-261639_at) (Athena Results)

TF sites	P value	#P	#S
MYB1AT	0.0033	46	122
CARGCW8GAT	0.0049	37	134
ARF binding site motif	0.0104	24	34
CACGTGMOTIF	0.0153	13	28
CCA1 binding site motif	0.0193	19	21
BoxII promoter motif	0.0234	26	36
Ibox promoter motif	0.0228	24	34
T-box promoter motif	0.0236	31	48

Table 4.5: TF sites of At4g14960 (probe ID-245270_at) (Athena Results**)**

TF sites	P value	#P	#S
DRE core motif		1	2

ABRE-like binding site motif		1	3
AG binding site motif	0.0010	1	2
AGL1ATCONSENSUS	0.0028	1	2
Ibox promoter motif		1	2
ACGTABREMOTIFA2OSEM		1	1
AGATCONSENSUS	0.0107	1	2
ATHB6 binding site motif		1	1
BoxII promoter motif		1	1
CACGTGMOTIF		1	4
CARGCW8GAT		1	2
CArG promoter motif		1	2
DREB1A/CBF3		1	1
GADOWNAT		1	1
MYB1AT		1	1
MYB2 binding site motif	0.0136	1	1
MYB2AT		1	2
MYB4 binding site motif		1	1
T-box promoter motif		1	1
TATA-box motif		1	6

5. At5g62690 (1 probe) β-tubulin 1

Table 5.1: FatiGO+ analysis of **At5g62690** co-regulated genes
Genes in list1 used in analysis: 51
Genes in list2 used in analysis: 28757

**NO SIGNIFICANT TERMS**		

Table 5.2: Common TF sites of At5g62690 co-regulated genes (probe ID-247442_s_at) (Athena Results)

**TF sites**	**P value**	**#P**	**#S**
MYB2AT	0.0030	21	22
AtMYC2 BS in RD22	0.0043	24	34
MYCATERD1	0.0043	24	34
ARF binding site motif	0.0077	24	27
GAREAT	0.0287	31	51
MYB1AT	0.0298	43	94
MYB4 binding site motif	0.0497	38	66

Table 5.3: TF sites of At5g62690 (probe ID-247442_s_at) (Athena Results)

AtMYC2 BS in RD22		1	3
CARGCW8GAT		1	2
GAREAT		1	3
MYB binding site promoter		1	1
MYB1AT		1	2
MYB4 binding site motif		1	3

MYCATERD1		1	3
TATA-box motif		1	1
W-box promoter motif		1	4
