# Supplementary material for: A plant natriuretic peptide-like molecule of the pathogen Xanthomonas axonopodis pv. citri causes rapid changes in the proteome of its citrus host
Source: BMC Plant Biol. 2010 Mar 21;10:51. doi: 10.1186/1471-2229-10-51 (PMC2923525; doi:10.1186/1471-2229-10-51)
Supplement: Additional file 2 — Stimulus and mutants analysis of Arabidopsis thaliana homologues of the proteins identified in the proteomics assay. (A) Stimulus response analysis in Genevestigator and (B) Identification of mutants in which the Arabidopsis homologues of the identified citrus proteins encoding genes were transcriptionally up- or down-regulated. [file 1471-2229-10-51-S2.PDF]

Additional file 2: Stimulus and mutants analysis of *Arabidopsis thaliana* homologues of the proteins identified in the proteomics assay.

Genevestigator analysis

(A) Stimulus analysis

1. At2g39730 - 245061_at Rubisco activase

**Stimulus (genes down)**	**Log 2 ratio**
ABA_4 (+)	-5.89
ABA_5 (+)	-5.85
Dark 4 (Col-O)	-3.66
Cell sorting and protoplasting	-3.40
ABA_3 (+)	-3.23
Iron deficiency 2 (late)	-2.83
Norflurazon (Col-O)	-2.01
Nitrate low	-1.96
Drought 2 (Col-O)	-1.81
Night extension (late)	-1.74
**Stimulus (genes up)**	**Log 2 ratio**
Light 5 (AS-hyg)	3.57
Light 4 (AS-hyg)	2.57
BL/H3BO3 (+)	2.32
Light 2	2.28
Light 5 (cli186)	1.85
Heat_roots	1.58
Far red light	1.42
White light	1.34
Hypoxia 3 (04%; 48h)	1.3
Light 4 (cli186)	1.29

2. AtCg00120 (245024_at) ATP synthase CF1 α subunit

**Stimulus (genes down)**	**Log 2 ratio**
Norflurazon 2 (gun1-9)	-4.29
CaLCuV	-2.17
ABA_4 (+)	-1.69
Nitrate low	-1.68
Norflurazon	-1.62
Dark 4 (Col-O)	-1.53
ABA_3 (+)	-1.39
ABA_5 (+)	-1.34
Photoperiod:shift SD to LD (Ler; 9d)	-1.29
Zearalenone (+)	-1.26
**Stimulus (genes up)**	**Log 2 ratio**
Iron deficiency (LZ3)	2.76
Cell sorting and protoplasting	2.16

Isoxaben (+)	1.87
Hydrogen peroxide (+)	1.86
Hypoxia 3 (04%; 48h)	1.49
Cold_roots_early	1.46
Genotoxic_green_early	1.18
Nitrate(45mM)_sucrose(90mM)	1.10
Iron deficiency (LZ1)	1.09
BL_2(+)	1.08

3. AtCg00040 (245048_at) Maturase K

**Stimulus (genes down)**	**Log 2 ratio**
Norflurazon 2 (gun1-9)	-2.88
Nitrate low	-2.71
CaLCuV	-2.38
ABA_4 (+)	-1.69
Temp: shift 16C to 25C (Col; 5d)	-1.41
Hypoxia 3 (01%; 2h)	-1.39
Drought (dor)	-1.39
Iron deficiency 2 (late)	-1.33
Hypoxia 3 (04%; 2h)	-1.25
Elicitor: HrpZ (4h)	-1.18
**Stimulus (genes up)**	**Log 2 ratio**
Cell sorting and protoplasting	5.90
Hydrogen peroxide (+)	1.53
Nitrate(45mM)_sucrose(90mM)	1.50
Hypoxia 3 (04%; 48h)	1.42
Nematode 1(+)	1.31
2, 4 dichlorophenoxyacetic acid (+)	1.30
Lincomycin_2 (+)	1.24
Nitrate(45mM)_sucrose(90mM)	1.04
Nematode2_late (+)	0.99
Heat_roots	0.99

4. At4g14960 (2 probes)
4.1 At4g14960 - 245270_at Tubulin α-chain

**Stimulus (genes down)**	**Log 2 ratio**
Drought (wt)	-2.1
Light/Drought (aox1a(sail))	-2.04
Osmotic_green_late	-1.72
Heat_2	-1.49
Light 5 (cli186)	-1.47
Rotenone (2h)	-1.47
Light/Drought (aox1a(salk))	-1.37
Hypoxia (+)	-1.28
Salt_roots_late	-1.27
PCD: senescence	-1.23

Stimulus (genes up)	Log 2 ratio
Zeatin 2 (+)	1.07
Zeatin 3 (+)	1.05
Sucrose 2 (AS-hyg)	1.00
Nitrate(0mM)_sucrose(90mM)	0.91
Nitrate(0mM)_sucrose(30mM)	0.87
Glucose 2-4-6h	0.84
CO2 high	0.70
TuMV (zone2)	0.64
Nematode1 (+)	0.53
Dark 4 (csn5)	0.49

4.2 At4g14960 - 261639_at Tubulin α-chain

Stimulus (genes down)	Log 2 ratio
Light/Drought (aox1a(sail))	-3.91
Light/Drought (aox1a(salk))	-3.39
Drought (wt)	-2.73
CaLCuV	-2.45
Heat 2	-2.40
Rotenone (12h)	-2.17
PCD: senescence	-2.16
PAC (+)	-1.93
P.syringae 3(+)	-1.81
Hypoxia (+)	-1.45
**Stimulus (genes up)**	**Log 2 ratio**
Sucrose 2 (AS-hyg)	1.03
CO_2 high	0.97
Glucose 2-4-6h	0.95
Nematode1 (+)	0.88
Hypoxia 3 (04%; 48h)	0.82
Zeatin 3 (+)	0.81
Zeatin 2 (+)	0.76
Isoxaben (+)	0.72
Hypoxia 3 (01%; 48h)	0.67
Nitrate(0mM)_sucrose(90mM)	0.67

5. At5g62690 - 247442_s_at β-tubulin 1

Stimulus (genes down)	Log 2 ratio
PCD: senescence	-2.00
CaLCuV	-1.58
P.syringae 3(+)	-1.53
Drought 2 (Col-O)	-1.45
Light/Drought (aox1a(sail))	-1.35
Cycloheximide (+)	-1.06
Osmotic_green_late	-1.05
Light 5 (cli186)	-1.01

Benzothiadiazole 3 (mkk1)	-1.01
Light/Drought (aox1a(salk))	-0.93
**Stimulus (genes up)**	**Log 2 ratio**
Isoxaben (+)	1.57
Hypoxia 3 (01%; 48h)	0.66
ABA_5 (+)	0.65
CS 2 shoot	0.64
ABA_3 (+)	0.58
ABA_4 (+)	0.58
Zeatin 3 (+)	0.56
Night extension (late)	0.53
Cell sorting and protoplasting	0.52
Zeatin 2 (+)	0.48

(B) Mutant analysis

1. At2g39730 - 245061_at Rubisco activase

Mutation (genes down)	Log 2 ratio
ahg1-1	-2.66
ahg3-1	-2.61
brx	-1.65
LEC1-OXi	-1.62
csn4-1	-1.47
myb50.2	-1.11
slr-1	-1.10
csn5(csn5a-2 csn5b)	-1.09
myb50	-0.99
myb61.3	-0.86
**Mutation (genes up)**	**Log 2 ratio**
lec1-1.3	2.21
agl66	1.97
oxt6:AtCPSF30	1.54
agl104	1.33
agl18	1.20
agl29	1.19
ga1-3.1	1.13
aba1-1.2	0.91
BLS::TCP4	0.86
abi1-1.2	0.80

2. AtCg00120 (245024_at) ATP synthase CF1 α subunit

Mutation (genes down)	Log 2 ratio
mkk1/mkk2	-3.84
abh1.1	-1.84
lec1-1.2	-1.56
abh1	-1.38
LEC1-OXi	-1.32
agl66/104	-1.25
brx	-1.10
abh1.2	-0.95
mkk2	-0.82
Umkirch-1/Umkirch-3	-0.64
**Mutation (genes up)**	**Log 2 ratio**
lec1-1.3	2.85
arf6:arf8	2.54
oxt6:AtCPSF30	2.02
Mir-0	1.99
Se-0	1.81
ZAT12.1	1.69
Hh-0	1.66

Bla-1	1.57
ANAC092OE	1.53
gi11.2	1.36

3. AtCg00040 (245048_at) Maturase K

**Mutation (genes down)**	**Log 2 ratio**
oxt6:AtCPSF30	-2.29
LEC1-OXi	-1.88
hen1-1	-1.02
Brx	-0.95
mkk1/mkk2	-0.84
slr-1	-0.73
hsf1:hsf3.1	-0.7
lec1-1.2	-0.66
sol2	-0.64
pho1	-0.63
**Mutation (genes up)**	**Log 2 ratio**
lec1-1.3	4.07
lec1-1.4	2.85
lec1	2.02
MYB61.2	1.60
MYB61	1.38
Mir-0	1.37
Se-0	1.34
myb61.3	1.28
csn4-1	1.21
MYB61.1	1.15

4. At4g14960 (2 probes) Tubulin α-chain
4.1 At4g14960 - 245270_at

**Mutation (genes down)**	**Log 2 ratio**
Bla-1	-3.47
ga1-3.1	-1.97
dor	-1.81
35S::amiR-white-2(MIR172a)	-1.60
immutans	-1.50
35S::amiR-white-1(MIR172a)	-1.32
ARR22ox.2	-1.04
mpk4:ctr1	-0.85
LEC1-OXi	-0.79
mpk4	-0.78
**Mutation (genes up)**	**Log 2 ratio**
lec1-1.3	3.04
oxt6:AtCPSF30	1.88
Hh-0	1.33
mkk1	1.26

myb50.1	1.18
aba1-1.2	1.04
mkk2	1.00
myb50	0.95
Se-0	0.95
Mir-0	0.82

4.2 At4g14960 - 261639_at Tubulin α-chain

**Mutation (genes down)**	**Log 2 ratio**
35S::amiR-white-2(MIR172a)	-2.71
sfr3.1	-2.67
ga1-3.1	-2.51
sfr3	-2.35
35S::amiR-white-1(MIR172a)	-2.16
sfr6.2	-2.10
sfr3.2	-2.07
sfr6.3	-2.05
dor	-1.81
immutans	-1.73
**Mutation (genes up)**	**Log 2 ratio**
lec1-1.3	2.48
myb50.1	2.38
myb50	2.17
myb50.2	1.78
oxt6:AtCPSF30	1.77
mkk1	1.44
Bla-1	1.22
sph1.2	1.02
HIC	0.94
mkk2	0.87

5. At5g62690 - 247442_s_at β-tubulin 1

**Mutation (genes down)**	**Log 2 ratio**
35S::amiR-white-2(MIR172a)	-1.73
35S::amiR-white-1(MIR172a)	-1.37
immutans	-1.08
dor	-1.07
pvip1:pvip2	-1.02
ein2	-0.88
ARR22ox.2	-0.88
ga1-3.1	-0.79
agl65/66/104	-0.64
nahG	-0.62
**Mutation (genes up)**	**Log 2 ratio**
mkk1	2.07
oxt6:AtCPSF30	2.02

lec1-1.3	1.64
mkk2	1.52
mkk1/mkk2	1.04
sph1.2	0.70
ga1	0.62
gh3.5-1D	0.57
aba1-1.2	0.51
sfr2.3	0.48
